# Characterization of hospital and community-acquired respiratory syncytial virus in children with severe lower respiratory tract infections in Ho Chi Minh City, Vietnam, 2010

**DOI:** 10.1111/irv.12307

**Published:** 2015-04-23

**Authors:** Tran Anh Tuan, Tran Tan Thanh, Nguyen thi Thanh Hai, Le Binh Bao Tinh, Le thi Ngoc Kim, Lien Anh Ha Do, Nguyen thi Thuy Chinh B'Krong, Nguyen thi Tham, Vu thi Ty Hang, Laura Merson, Jeremy Farrar, Tang Chi Thuong, Menno D de Jong, Constance Schultsz, H Rogier van Doorn

**Affiliations:** a Children's Hospital 1Ho Chi Minh City, Vietnam; b Oxford University Clinical Research Unit in partnership with the Hospital for Tropical DiseasesHo Chi Minh City, Vietnam; c Nuffield Department of Medicine, Centre for Tropical Medicine, University of OxfordOxford, UK; d Department of Medical Microbiology, Academic Medical Centre, University of AmsterdamAmsterdam, The Netherlands; e Department of Global Health-Amsterdam Institute of Global Health and Development, Academic Medical Centre, University of AmsterdamAmsterdam, The Netherlands

**Keywords:** nosocomial infection, phylogenetics, respiratory syncytial virus

## Abstract

**Background:**

Human respiratory syncytial virus (RSV) is an important community and nosocomial pathogen in developed countries but data regarding the importance of RSV in developing countries are relatively scarce.

**Methods:**

During a 1-year surveillance study in 2010, we took serial samples from children admitted to the Emergency Unit of the Respiratory Ward of Children's Hospital 1 in Ho Chi Minh City, Vietnam. RSV was detected within 72 hours of admission to the ward in 26% (376/1439; RSV A: *n* = 320; RSV B: *n* = 54; and RSV A and B: *n* = 2). Among those negative in the first 72 hours after admission, 6·6% (25/377) acquired nosocomial RSV infection during hospitalization (RSV A: *n* = 22; and RSV B: *n* = 3).

**Results:**

Children with nosocomial RSV infection were younger (*P* = 0·001) and had a longer duration of hospitalization (*P* < 0·001). The rate of incomplete recovery among children with nosocomial RSV infection was significantly higher than among those without (*P* < 0·001). Phylogenetic analysis of partial G gene sequences obtained from 79% (316/401) of positive specimens revealed the co-circulation of multiple genotypes with RSV A NA1 being predominant (A NA1: *n* = 275; A GA5: *n* = 5; B BA3: *n* = 3; B BA9: *n* = 26; and B BA10: *n* = 7). The RSV A GA5 and RSV B BA3 genotypes have not been reported from Vietnam, previously.

**Conclusion:**

Besides emphasizing the importance of RSV as a cause of respiratory infection leading to hospitalization in young children and as a nosocomial pathogen, data from this study extend our knowledge on the genetic diversity of RSV circulating in Vietnam.

## Introduction

Human respiratory syncytial virus (RSV) is a negative-sense, non-segmented single-stranded RNA virus belonging to the subfamily of *Pneumovirinae* in the family of *Paramyxoviridae*. RSV is a highly contagious respiratory pathogen and a leading cause of hospitalization among infants and young children in both developing and developed countries.^1^–^3^ The typical clinical syndrome of RSV infection in hospitalized children is bronchiolitis; children are usually under 2 years of age and severity may vary from relatively mild respiratory illness to severe respiratory compromise. It is estimated that almost all children have been infected by RSV at least once during the first 2 years of life.^4^ Infections with RSV occur in predictable intervals, during winter months in temperate climate regions and rainy months in tropical climate regions.^2^,^5^–^7^

Respiratory syncytial virus is also recognized as an important nosocomial pathogen in developed countries. During epidemics, nosocomial RSV infections were reported in up to 32% of children who were admitted to the hospital for a non-RSV illness.^8^ Children with nosocomial RSV infection are usually symptomatic with manifestations of lower respiratory tract involvement. Particularly, children with underlying conditions such as prematurity, chronic or congenital heart and pulmonary diseases, immunosuppression and in general those who are hospitalized for a prolonged period of time are at risk for nosocomial RSV infection.^9^–^11^ However, data regarding the importance of RSV as a nosocomial pathogen from developing countries are scarce^12^ and no such data are available from Vietnam, yet.

The genome of RSV encodes 10 different functional proteins: non-structural protein 1 (NS1) and 2 (NS2); nucleocapsid proteins N, P, and L; matrix protein M and M2; small hydrophobic protein (SH); attachment glycoprotein (G); and fusion protein (F). The G and F proteins are the major antigenic determinants, and the nucleotide sequences encoding these are also the most variable in the genome.^13^,^14^ Based on antigenic and genetic variations, RSV has been classified into two major antigenic subgroups: A and B, which can be further divided into genotypes based on the nucleotide sequences of the G and/or F genes.^5^,^15^ The G protein contains two hypervariable regions (HVR), and the nucleotide sequence of the second HVR at the C terminus (HVR2) is commonly used in genetic characterization and genotyping.^5^,^15^ Currently, there are 10 genotypes within subgroup A: GA1 to GA7, SAA1, NA1, and NA2. Within subgroup B, there are at least 20 genotypes: GB1 to GB4, BA1 to BA10, URU1, URU2, and SAB1 to SAB4.^5^ Both RSV subgroups A and B are co-circulating worldwide, but subgroup A viruses are more frequently detected from clinical diagnostic specimens than subgroup B viruses.^7^,^16^,^17^

Here, we describe the clinical characteristics of RSV infection in children admitted to the Emergency Unit of the Respiratory Ward in Children's Hospital 1, a large pediatric referral hospital in Ho Chi Minh City during one RSV season between January and December 2010, distinguishing between community-acquired and nosocomial infection. In addition, we sequenced part of the RSV G gene and genotyped 316/401 (79%) RSV-positive patients. Molecular characterization of RSV detected in our study contributes to the limited available data of the genetic diversity of RSV circulating in Vietnam.

## Materials and methods

### Study design

Data presented here are part of a prospective, non-randomized, crossover intervention study comparing the incidence of nosocomial RSV infection in two rooms of a single emergency unit (EU) when applying basic or enhanced infection control procedures. This intervention study was prematurely discontinued after an interim analysis showed that the nosocomial infection rate was lower than expected, similar on the two wards and the required patient numbers to maintain power were not feasible to achieve.

This study was conducted at the EU of the respiratory ward of Children's Hospital 1 (CH1) in Ho Chi Minh City (HCMC), Vietnam. CH1 is one of the two large pediatric referral hospitals for southern Vietnam. The respiratory ward has two suites of 11 rooms with a total of 80 beds including the EU, which is composed of two rooms at the end of the suite and has a total of 25 beds. Children admitted to the EU are those in severe conditions with evidence of consolidation on chest X-ray, tachypnea (infant up to 12 months ≥60; children 1–6 years ≥40; and children 6–12 years ≥30), and peripheral arterial oxygen saturation (SpO_2_) ≤92% requiring respiratory support (nasal cannula, face mask, nasal continue positive pressure, or ventilation).

### Sample collection

All children (<16 years of age) admitted to the EU between January and December 2010 were eligible and were enrolled unless they or their legal guardian were unwilling to provide respiratory samples. Clinical, demographical, and basic laboratory data were collected and documented by study clinicians in case report forms (CRFs). For diagnosis of community-acquired RSV infection, flocked nasopharyngeal swabs (diagnostic swabs) were obtained from all children on admission to the EU and at 72 hours after admission. Seventy-two hours were chosen based on estimated incubation times of RSV of between 2 and 8 days, but most frequently 4–6 days. For monitoring nosocomial RSV infection, additional flocked swabs (screening swabs) were taken twice weekly from all enrolled patients on the EU on Mondays and Thursdays. Swabs were collected in 1 ml of viral transport medium (VTM) and stored at 4°C pending daily transportation to the Molecular Diagnostic Laboratory of Oxford University Clinical Research Unit in HCMC, where they were stored at −80°C until further testing.

### Ethics

The study was approved by the Scientific and Ethical Committee of CH1 and by the Oxford University Tropical Research Ethical Committee (OxTREC). The need for written informed consent from each patient was waived on the basis of this being a minimal risk service evaluation of hospital infection control procedures.

### Diagnostic testing

Viral RNA was extracted from 100 μl of nasopharyngeal specimens using the MagNA Pure 96 nucleic acid extraction system (Roche Diagnostics, Basel, Switzerland) following the manufacturer's instructions. Viral RNA was eluted in 60 μl elution buffer and 5 μl viral RNA was used in the diagnostic and subtyping real-time RT-PCR for RSV A or B infection as described previously.^18^ The remaining viral RNA was stored at −80°C until further analyses. Diagnosis for infections caused by other viral and bacterial pathogens was not performed.

### RT-PCR and sequencing

HVR2 of the G protein gene of both subgroups A and B viruses was amplified directly from clinical specimens using semi-nested RT-PCRs. The external RT-PCR, universal for both subgroups A and B viruses, was performed using the SuperScript II Onestep RT-PCR Kit (Invitrogen, Carlsbad, CA, USA) according to the manufacturer's instructions with the addition of the forward primer ABG490 and the reverse primer F164 (Table1) and 5 μl viral RNA.^19^ The thermal cycling conditions were 50°C for 30 minutes, 95°C for 2 minutes, followed by 40 cycles of 94°C for 20 seconds, 50°C for 1 minute, and 72°C for 45 seconds, and a final extension at 72°C for 7 minutes. One microliter of the RT-PCR products was used in the semi-nested PCR using HotStar Taq Polymerase (Qiagen, Hilden, Germany) according to the manufacturer's instructions with the addition of the forward subgroup A-specific primer AG655 or subgroup B-specific primer BG517 and the same reverse primer F164 (Table1). The thermal cycling conditions were 95°C for 15 minutes, followed by 40 cycles of 94°C for 20 seconds, 55°C for 30 seconds, and 72°C for 45 seconds, and a final extension at 72°C for 7 minutes. PCR products were subjected to electrophoresis in 2% agarose gel; these were checked for the presence of 450-/585-bp amplicons of subgroup A/B viruses, respectively. PCR products were purified by ethanol precipitation and subsequently diluted in 25 μl molecular grade water prior to sequencing analysis. Approximately 20 ng of PCR amplicons were used in each sequencing reaction using the ABI BigDye Terminator cycle sequencing kit on the ABI 3730XL automatic DNA analyzer (Applied Biosystems Inc., Foster City, CA, USA) following the manufacturer's instructions.

**Table 1:** Primers used for amplification and sequencing the second hypervariable region of the G gene protein of respiratory syncytial virus (RSV)^19^

Name	Sequence	Corresponding gene and nucleotide	Reference strain
ABG490	ATGATTWYCAYTTTGAAGTGTTC	G protein gene, nucleotide 497–519	RSV A prototype strain A2 (GenBank Accession Number M11486)
F164	GTTATGACACTGGTATACCAACC	F protein gene, nucleotide 164–186	RSV A prototype strain A2
AG655	GATCYCAAACCTCAAACCAC	G protein gene, nucleotide 655–674	RSV A prototype strain A2
BG517	TTYGTTCCCTGTAGTATATGTG	G protein gene, nucleotide 517–538	RSV B prototype strain CH18537 (GenBank Accession Number M17213)

### Sequence analysis

Sequences were assembled using ContigExpress software (Vector NTI Suite 7.1; Life Technologies, Invitrogen) and aligned with reference sequences of RSV A and B subgroups retrieved from GenBank (Table S1) using BioEdit software v7.0.1 (Isis Pharmaceuticals Inc., Carlsbad, CA, USA). Phylogenetic trees were constructed in MEGA software v5.05 (www.megasoftware.net) using the Tamura–Nei nucleotide substitution model with 1000 bootstrap replicates. The pairwise nucleotide and amino acid distances (p-distances) were estimated using the integrated pairwise calculator of the MEGA software. The deduced amino acid sequences of HVR2 of the G protein of RSV were generated using BioEdit software. Potential *O-* and *N*-glycosylation sites were predicted by the presence of specific glycosylation motifs such as NXT (where X is not a proline) for *N*-glycosylation and KPT, TTKK, and TTKT motifs for *O-*glycosylation.^5^,^17^,^19^,^20^

### Data analysis

Children with a positive RSV RT-PCR on admission or 72 hours after admission were defined to have community-acquired RSV infection. Those who tested negative on admission and after 72 hours but had a positive RSV RT-PCR in subsequent nasopharyngeal swabs were defined as having nosocomial RSV infection.^12^ Statistical analyses were performed using SPSS v19.0 (SPSS, Inc., Chicago, IL, USA) in which categorical variables were compared using chi-square or Fisher's exact tests and continuous variables were compared using Mann–Whitney U-test. Both univariate and multivariate analyses were performed using R v3.0.2 (www.r-project.org). The multivariate analyses for the effects of several covariates on the binary outcomes recovery and other than full recovery and on the continuous outcome duration of hospitalization were carried out using multivariate logistic regression and linear regression analysis, respectively. The covariates were as follows: nosocomial RSV, age, birth weight, prematurity, admitted in previous 14 days, transfer and pre-existing medical condition. All statistical analyses were performed at two-sided 5% significance level.

## Results

### Patient characteristics

Between January and December 2010, 1439 patients were enrolled. The median length of stay was 4 days (interquartile range [IQR]: 2–8 days). The median age of the children was 22·3 weeks (IQR: 9·6–49·0). The median weight at birth was 2·9 kg (IQR: 2·5–3·2), and 22·4% (322/1435) of children had pre-existing diseases (see Table3 note). A history of premature birth was documented in 19·3% cases (278/1439). One-third of the children (492/1439) were referred from other wards. Twelve percent of the children (169/1439) were transferred from the EU to PICU, other wards or other specialized hospitals. Thirty-seven children died during hospital admission, but no follow-up after transfer to other hospital or discharge home was included in the study protocol, and therefore, this may be an underestimate.

### RSV detection

**Figure 1:**
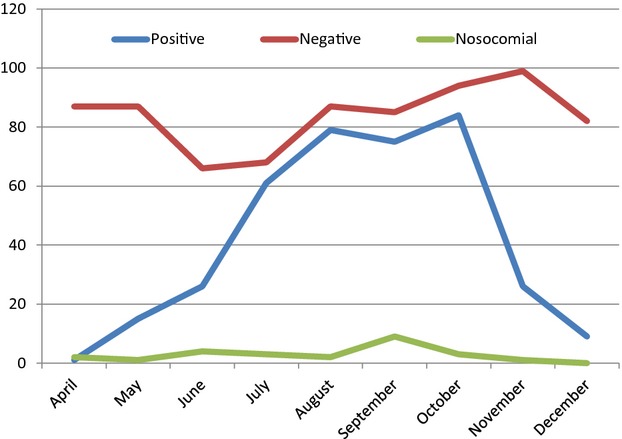
The distribution of community and nosocomial RSV cases in Ho Chi Minh City, Vietnam, during April–December 2010.

Respiratory syncytial virus infection was diagnosed by viral RNA detection in respiratory specimens of 376/1439 admitted children (26%) of whom 363 were positive on admission and an additional 13 within the first 72 hours after admission to the emergency unit. Among the 376 positive children, 320 were infected with RSV subgroup A, 54 with RSV subgroup B, and two with both RSV subgroups. RSV was not detected during the first 3 months (January–March) of 2010, while high numbers (>50 per month) were detected during July–October (Figure1). The baseline characteristics of all children, stratified for RSV positivity or negativity within the first 72 hours after admission, are displayed in Table2. Baseline characteristics excluding transferred patients are displayed in Table3.

**Table 2:** Demographic and clinical characteristics of respiratory syncytial virus (RSV)-positive versus RSV-negative patients at enrollment

Characteristics (%)	RSV negative (*n* = 1063)	RSV positive (*n* = 376)	*P* value
Baseline information			
Male (%)	62·5 (660/1056)	62·7 (232/370)	0·948
Age (weeks, median)	All patients: 22·3 (9·6–49·0)		
	24 (10–54·3)	17·9 (8·6–37·5)	0·000
Birthweight (kg)	2·9 (2·5–3·1)	3·0 (2·6–3·3)	0·003
Preterm birth (<37 weeks) (%)	20·5 (218/1062)	16·0 (60/376)	0·057
Previous hospitalization (%)	30·4 (319/1049)	21·3 (80/376)	0·000
Within previous 14 days (%)	19·9 (212/1063)	16·8 (63/376)	0·202
Transfer from other ward (%)	36·9 (392/1063)	27·1 (102/376)	0·001
Pre-existing illness (%)	24·9 (264/1061)	15·5 (58/374)	0·000
Clinical information			
Fever (%)	68·6 (729/1063)	72·3 (272/376)	0·172
Runny nose (%)	36 (383/1063)	69·4 (261/376)	0·000
Pulse (/minute, median)	140 (136–160)	140 (136–150)	0·022
Respiratory rate (/minute, median)	56 (52–60)	58 (54–60)	0·031
Arterial oxygen saturation (SpO_2_) (median)	89 (87–90)	90 (88–90)	0·000
Crackles (%)	89·5 (950/1061)	98·4 (370/376)	0·000
Wheeze (%)	48·2 (510/1058)	74·4 (279/375)	0·000
Altered consciousness (%) (agitated or lethargic)	69·9 (741/1060)	77·9 (293/376)	0·003
Admission diagnosis			
Pneumonia (%)	75·3 (800/1063)	51·1 (192/376)	0·000
Bronchiolitis (%)	19·8 (211/1063)	48·1 (181/376)	0·000
Mechanical ventilation (%)	3·9 (41/1030)	2·6 (8/370)	0·136
Duration of hospitalization (days)	4·0 (2–9)	4·0 (2–6)	0·285
Outcome other than full recovery (%)	13·2 (140/1063)	7·7 (29/376)	0·005
Deaths (%)	24·3 (34/140)	10·3 (3/29)	0·138
Chest X-Ray			
Consolidation (%)	51·8 (550/1062)	37·1 (139/375)	0·000
Peribronchial cuffing (%)	56·5 (601/1063)	64·3 (241/375)	0·01
Airbronchogram (%)	48·1 (511/1063)	37·3 (140/375)	0·000
Laboratory findings			
Hemoglobin (g/dl)	10·7 (9·8–11·5)	10·5 (9·8–11·3)	0·019
Hematocrit (%)	32·5 (30·1–35·5)	32·2 (29·9–34·8)	0·051
Leukocytes (×1000/μl)	14 (10·3–16·5)	10·9 (8·5–14·5)	0·000
Neutrophils (%)	54·7 (35·6–64·1)	39·4 (28·5–55)	0·000
Lymphocytes (%)	37 (26·6–53·2)	52·3 (36·4–62)	0·000
Platelets (×1000/μl)	311 (210–415)	348 (280·3–413)	0·000

**Table 3:** Demographic and clinical characteristics of respiratory syncytial virus (RSV)-positive and RSV-negative children who were directly admitted from the community and who had not been hospitalized in the 14 days prior to admission

Characteristics (%)	RSV negative (*n* = 623)	RSV positive (*n* = 254)	*P* value
Baseline information (excluding referred and 14 days discharged patients)			
Male (%)	60·3 (373/619)	64 (160/250)	0·318
Age (weeks, median)	25·1 (9·9–52·6)		
	28·3 (11·4–59·4)	17·9 (8·5–41·2)	0·000
Birthweight (kg)	2·9 (2·6–3·2)	3·1 (2·7–3·3)	0·000
Preterm birth (<37 weeks) (%)	16·7 (104/623)	12·6 (32/254)	0·150
Previous hospitalization (%)	4·2 (26/612)	2·4 (6/248)	0·235
Pre-existing illness (%)	18·1 (113/623)	12·2 (31/254)	0·035
Clinical information			
Fever (%)	72·7 (453/623)	72 (183/254)	0·868
Runny nose (%)	41·6 (259/623)	75·6 (192/254)	0·000
Pulse (/minute, median)	140 (132–148)	140 (134–142)	0·121
Respiratory rate (/minute, median)	58 (54–60)	58 (54–60)	0·7
Arterial oxygen saturation (SpO_2_) (median)	89 (88–90)	90 (88–90)	0·000
Crackles (%)	90·2 (562/623)	96·5 (245/254)	0·000
Wheeze (%)	54 (336/622)	81·5 (207/254)	0·000
Altered consciousness (%) (agitated or lethargic)	70·2 (436/621)	74·0 (188/254)	0·248
Admission diagnosis			
Pneumonia (%)	70·5 (439/623)	42·1 (107/254)	0·000
Bronchiolitis (%)	18·5 (151/263)	56·7 (144/254)	0·000
Mechanical ventilation (%)	2·3 (14/605)	1·6 (4/251)	0·609
Duration of hospitalization (days)	3 (2–6)	3 (2–5)	0·259
Outcome other than full recovery (%)	8·8 (55/623)	6·3 (16/254)	0·274
Deaths (%)	1·9 (12/623)	1·2 (3/254)	0·573
Chest X-Ray			
Consolidation (%)	42·9 (267/623)	31·2 (79/253)	0·001
Peribronchial cuffing (%)	61·2 (381/623)	68·4 (173/253)	0·045
Airbronchogram (%)	38·2 (238/623)	31·6 (80/253)	0·075
Laboratory tests			
Hemoglobin (g/dl)	10·9 (10·1–11·5)	10·6 (9·9–11·4)	0·021
Hematocrit (%)	32·9 (30·5–35·5)	32·6 (30·0–34·9)	0·072
Leukocytes (×1000/μl)	14·2 (10·7–16·5)	10·9 (8·5–14)	0·000
Neutrophils (%)	58·5 (39·7–65)	39·1 (27·4–55·1)	0·000
Lymphocytes (%)	35·1 (26·1–49·9)	53·6 (36·6–63·6)	0·000
Platelets (×1000/μl)	297 (208–388)	342 (270·5–402·5)	0·000

Pre-existing diseases include a broad range of diseases such as Down's syndrome, cerebral palsy, pneumocephaly, brain tumor, asthma, laryngomalacia, tracheal stenosis, cleft palate, epilepsy, tetralogy of Fallot, severe malnutrition, astrocytoma, retinopathy of prematurity, pediatric bipolar disorder, cholestatic jaundice, diaphragmatic hernia, esophageal atresia, gastroesophageal reflux, Hurler syndrome, hydrocephaly, lymphangioma, jejunal atresia, meningitis, myasthenia, neuromuscular disease, osteochondrodysplasia, osteopetrosis, Pierre–Robin syndrome, Wedrnig-Hoffman disease.

Outcome other than full recovery includes children in critical condition who were transferred to specialized hospitals/wards or death.

Among those negative for RSV on admission and after 72 hours during the RSV season (April–December; 780/1156), 377/780 were admitted on EU longer than 72 hours and were screened for nosocomial RSV infection. RSV viral RNA was detected in specimens of 25 children (22 RSV subgroup A, 3 RSV subgroup B), indicating an overall rate of nosocomial infection on the emergency unit of 6·6% of total patients at risk (25/377) or 2·01 cases/patient years of admission. The median time from admission to the detection of nosocomially acquired RSV was 3 weeks (IQR: 2–4) and ranged from 2 to 24 weeks.

### Clinical presentation

Children with RSV were significantly younger (17·9 versus 24 weeks; *P* < 0·001) and had a higher documented birthweight (3·0 versus 2·9 kg; *P* = 0·001). Pre-existing medical conditions were documented mostly in children without RSV infection (264/1061 versus 58/374; *P* < 0·001). Most children had a clinical diagnosis of bronchiolitis (392/1439) or pneumonia (992/1439) on admission. Children with RSV were more likely to have a diagnosis of bronchiolitis (*P* < 0·001), whereas RSV negatives were more likely to have a diagnosis of pneumonia (*P* < 0·001) (Tables2 and 3). Two-thirds of children (1001/1439) were febrile, but this was not different among children with and without RSV infection. Runny nose and wheeze were more frequently reported in children with RSV infection (*P* < 0·001). Children with RSV infection had a higher median arterial oxygen saturation (SpO_2_) (90 versus 89; *P* < 0·001), and a significant difference in respiratory rates was observed (*P* = 0·031) (Tables2 and 3).

There was no difference in duration of hospital stay (4·0 versus 4·0 days; *P* = 0·285), but the proportion of children with an outcome other than full recovery was significantly lower among children with RSV (7·7% versus 13·2%; *P* = 0·005) (Tables2 and 3), but when corrected for pre-existing medical conditions (more frequent among RSV-negative children), no association was found. All above parameters were also compared among children with RSV subgroups A and B infections; no significant differences were found (data not shown), except that the initial viral load during RSV subgroup A infection was slightly lower than in RSV subgroup B infection (Cp value RSV A of 30·8 versus 29·1 of RSV B; *P* = 0·031).

Twenty-five children with nosocomial RSV infection were identified in this study (22 subgroup A and 3 subgroup B). Compared to children who remained negative for RSV during admission (*n* = 352), children with nosocomial RSV infection were significantly younger (9·0 versus 16·2 weeks; *P* = 0·001), the duration of hospital stay was much longer (21·0 versus 7·0 days; *P* < 0·001) (Table4), and the proportion of children with an outcome other than full recovery in children with nosocomial RSV infection was significantly higher than those without RSV (52 versus 17%; *P* < 0·001). These associations remained significant when corrected for age, weight at birth, prematurity, previous hospital admission, and pre-existing medical conditions.

**Table 4:** Factors associated with nosocomial respiratory syncytial virus (RSV) infection among 377 children screened for (RSV) infection >72 hours after admission

	Non-nosocomial (*n* = 352)	Nosocomial (*n* = 25)	*P* value
Median week from admission		3 (2–4)	
Male (%)	65·9 (230/349)	60·0 (15/25)	0·664
Age (weeks, median)	16·2 (8·8–43·5)	9·0 (6·5–14·7)	0·001
Preterm birth (<37 weeks)	26·8 (94/351)	44 (11/25)	0·103
Birthweight (kg)	2·7 (2·2–3·0)	2·5 (2·2–3·1)	0·113
Previous hospitalization (%)	40·2 (140/348)	56 (14/25)	0·143
Within previous 14 days (%)	28·1 (99/352)	40 (10/25)	0·370
Transfer from other ward (%)	46·9 (165/352)	60·0 (15/25)	0·220
Pre-existing illness (%)	34·8 (122/351)	32 (8/25)	0·832
Duration of hospitalization (days)	7·0 (5·0–13·0)	21·0 (12·0–30·0)	0·000
Outcome other than full recovery (%)	16·8 (59/352)	52·0 (13/25)	0·000
Deaths	2·6 (9/352)	16·0 (4/25)	0·007
Laboratory tests			
Hemoglobin (g/dl)	10·7 (9·8–11·5)	10·3 (9·2–11·5)	0·318
Hematocrit (%)	32·3 (30·1–35·3)	30·8 (27·4–33·5)	0·071
Leukocytes (×1000/μl)	14·0 (10·7–17·1)	13·0 (9·6–16·4)	0·313
Neutrophils (%)	49·5 (31·9–61·2)	32·0 (26·0–44·8)	0·001
Lymphocytes (%)	39·7 (28·1–56·1)	53·7 (45·7–65·2)	0·002
Platelets (×1000/μl)	311·0 (210·0–425·0)	268·0 (185·5–412·0)	0·230

### Molecular characteristics of RSV

Sequencing of HVR2 of the G gene was successful in a total of 316/401 (79%) RSV-positive specimens. These included 280 subgroup A viruses (267 community RSV [cRSV] and 13 nosocomial RSV [nRSV]) and 36 subgroup B viruses (35 cRSV and 1 nRSV). When directly comparing the subgroup A sequences, the maximum divergence at nucleotide and amino acid levels were 12·8% and 24·1%, respectively. The maximum divergence when compared to the prototype A2 strain was larger at both nucleotide (13·6%) and amino acid (27·8%) levels. The maximum divergence of subgroup B sequences was 8·6% at nucleotide level and 17·7% at amino acid level. Compared to the prototype strain CH18537, the maximum divergence at nucleotide and amino acid levels of subgroup B viruses were 14·5% and 26%, respectively.

**Figure 2:**
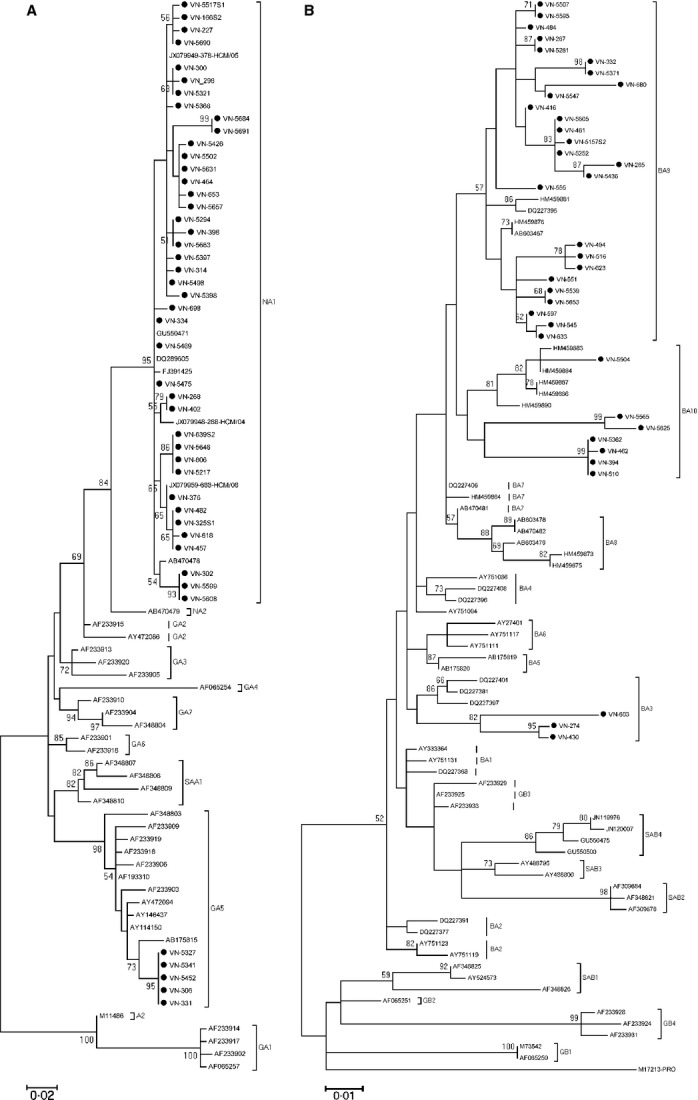
(A and B) Phylogenetic tree of representative Vietnamese RSV A and B from the nucleotide sequences of the C-terminal second hypervariable region of the G gene. Tree was constructed by MEGA 5.1 software using Tamura–Nei nucleotide substitution model with 1000 bootstrapping replicates. Bootstrap values greater than 50% are shown on the branch nodes. The Vietnamese strains from this study are indicated by solid rounds.

Phylogenetic tree analysis revealed that the 280 Vietnamese subgroup A viruses were clustered into two genotypes. A majority of subgroup A viruses (*n* = 275) clustered into the NA1 genotype and five viruses clustered into the GA5 genotype (Table S3) (Figure2A). The 36 Vietnamese subgroup B viruses clustered into three distinct genotypes: BA3 (*n* = 3), BA9 (*n* = 26; 25 cRSV and 1 nRSV), and BA10 (*n* = 7) (Figure2B). The 13 nRSV A viruses all belonged to the NA1 genotype, and among them six viruses shared the same sequence while seven viruses each had unique sequences (Table S2), suggesting multiple viruses were causing nosocomial infections in the EU during the study period. The six identical nRSV A sequences were identical to 114 sequences of the cRSV viruses. From the other 7, 5 nRSV viruses with a unique sequence were each identical to 1–15 sequences of the cRSV viruses. Two unique nRSV sequences were different from all the sequences of the cRSV viruses (including existing sequences in GenBank) by at least 1 and 5 nucleotides (Table S2). The sequence of the nRSV subgroup B virus belonged to the BA9 genotype and was also different from all the sequences of the cRSV subgroup B viruses by at least 1 nucleotide.

The Vietnamese NA1 viruses also possessed the previously reported NA1-specific amino acid residues Asp237 (98·5%), Leu274 (94·9%), Ser292 (100%), and a premature stop codon at position 298 (100%).^20^ Interestingly, the previously reported GA2-specific residues Thr269 and Ser289 were also found in all Vietnamese NA1 viruses (Table S3). The NA1-specific Asp237 residue was also found in all five Vietnamese GA5 viruses. Other amino acid substitutions specific for the GA5 genotype reported previously, including Pro241, Asn250, Ser251, Thr274, Ile279, Ile295, Asn297, and a stop codon at position 299, were also found in all the Vietnamese GA5 viruses.^5^,^17^

The Vietnamese RSV A viruses contained three potential *N*-glycosylation sites in HVR2 of G at positions 237, 250 or 251, and 294 as reported previously.^5^,^17^,^19^,^20^ The *N*-glycosylation site at position 237 was found in all five GA5 viruses and only in 3/275 NA1 viruses (Table S3). The second *N*-glycosylation site at position 251 was found in most of the Vietnamese NA1 viruses (Table S3). Similar to previous findings in GA5 viruses,^5^ the second glycosylation site at position 251 in the Vietnamese GA5 viruses was shifted to position 250 due to dual Gln250 and Ser251 mutations. The third glycosylation site at position 294 was conserved in all GA5 viruses but only found in a minority of NA1 viruses due to the Ile296 substitution. The *O-*glycosylation sites were highly conserved among RSV A viruses with the exception of the GA5 genotype, as observed previously among Cambodian RSV A viruses.^5^

The Vietnamese RSV B viruses were predicted to have a G protein length of between 282 and 319 amino acids (Table S4). The BA9 genotype was predominant among the Vietnamese subtype B viruses and all contained a dual stop codon at positions 313 and 320. All the BA10 genotype viruses contained the previously reported specific residue Gly292, but only 2/7 contained the Pro231, reported previously among most Cambodian BA10 viruses.^5^ The Vietnamese GA3 genotype viruses contained 3 potential genotype-specific residues: Pro222, Ile239, and Arg282 (Table S4). Two potential *N*-glycosylation sites (at positions 296 and 230) and one glycosylation site (at position 234) were, similar to the Cambodian B viruses, highly conserved among Vietnamese subgroup B viruses.^5^ The newly discovered *N*-glycosylation site at position 230 (as a consequence of a Ser231 substitution) in most Cambodian BA10 viruses^5^ was only found in 2/7 Vietnamese BA10 viruses (Table S4).

## Discussion

We describe the detection rates of community-acquired and nosocomial RSV infections between January and December 2010 in the Emergency Unit of the Respiratory Ward of Children's Hospital 1, Ho Chi Minh City, Vietnam. 376/1439 (26·1%) children had RSV detected within 72 hours after admission; all RSV cases were detected between April and December (376/1156 patients [32·5%]). In this period, the overall rate of nosocomial RSV infection in the EU was 6·6% (*n* = 25), which falls in the lower range of previously reported rates of nosocomial RSV infections (2·7–32%).^8^,^12^,^21^ This number is influenced by the fact that the duration of admission was less than 72 hours in the majority of children and these were not assessed for nosocomial RSV. The number may not reflect the true overall nosocomial infection rate, but rather the rate of infections detected after 72 hours of admission on the EU, as previous hospitalizations or longer duration of hospitalization due to transfers were not taken into account and potential cases of nRSV may thus have been classified as cRSV. The implementation of enhanced infection control measures in one study room and training on infection control measures for study staff in both rooms may also have had an effect on the reduction of transmission.^22^

Children with nosocomial RSV were younger, had a longer total duration of hospitalization and a lower rate of full recovery at discharge. Young age and duration of hospitalization are among the established risk factors for nosocomial RSV infection.^10^ Pre-existing medical conditions, also a proven risk factors for nosocomial RSV infection,^10^ were not found to be associated with nosocomial RSV risk in this study. This may have been due to the nature of the study site, where very young children in severe condition with a history of hospitalization and pre-existing illness are overrepresented.

During the season of study, RSV subgroup A was dominant. This is in accordance with data from the same epidemic season from Children's Hospital 1 (Do Lien Anh Ha, unpublished results) and Children's Hospital 2, HCMC.^7^ Surveillance studies among in- and outpatients in Cambodia (which shares a border with the southern part of Vietnam, about 100 km away from HCMC) from 2005 to 2009 revealed that subgroup B was dominant in 2005, 2008, and 2009.^5^

The dominant RSV A genotype was NA1. This genotype was newly identified in Niigata, Japan, during the 2004–2005 season and is considered a descendant of the GA2 genotype that was then circulating worldwide.^20^ Genotype NA1 has since disseminated and is now the most commonly detected RSV A genotype worldwide, including in Vietnam^7^ (Figure2). GA5 viruses were co-circulating, and these have not been reported from Vietnam, previously. During a 5-year surveillance (2005–2009) for RSV in Cambodia, GA5 was detected in 2007 as a non-dominant lineage.^5^

In accordance with previous work from Children's Hospital 2, HCMC,^7^ the BA9 and B10 genotypes of RSV subgroup B were also detected in this study. These genotypes were also first described in Niigata, Japan, in 2006^23^ and are descendants of the BA strains that were described in Buenos Aires, Argentina in 1999.^24^ Besides the BA9 and BA10 genotypes, the BA3 genotype was also detected in this study and this genotype has not been reported from Vietnam, previously.^7^ The BA3 strains were also first described in Buenos Aires, Argentina, but in 2002, and have been frequently detected in European countries during 2002–2005.^15^ However, BA3 strains have not been detected in recent surveillance studies from Spain, Japan, and Cambodia.^5^,^15^,^23^ A novel genotype (BA-C) was reported from China in 2008–2009,^25^ which is most closely related to BA3. The simultaneous regional and worldwide emergence, circulation, and disappearance of multiple genotypes and subtypes of RSV suggest there is global strain replacement potentially driven by fitness or antigenic advantage (although no associations with more severe disease or larger outbreaks have been found) and requires monitoring and surveillance to inform about emergence of novel—potentially more virulent—genotypes and future vaccine development efforts.

The resolution/discriminatory power of the 450-/585-bp-long sequences from the RSV A/B HVR2 region of the G gene did not allow for detailed assessment of transmission chains, as the majority of sequences were identical to groups of sequences that were detected throughout the RSV season. Among 25 nosocomial RSV viruses, three viral sequences were not identical to any of the community-acquired sequences. One was different by five nucleotides from the closest related virus, two by only one nucleotide. No discrepancies were seen in the (double bidirectional) sequencing results at this position. This may further be caused by either 1) unsuccessful sequencing of the transmitted community-acquired virus (sequencing successful in 316/401 RSV patients), 2) sequencing error introduced by reverse transcriptase, 3) transmission by a relative of healthcare worker, or 4) transmission of an undetected subpopulation (although the sequencing chromatograms did not show any double peaks at this position) or antigenic drift.

Our study confirms the importance of RSV as a pathogen in early childhood, accounting for 26% of all hospitalizations and 32·5% during the RSV season on a respiratory ward in 2010. Molecular data from this study revealed the co-circulation of different genotypes of subgroup A and B RSV during the 2010 epidemic season in Vietnam, in accordance with data on RSV circulation worldwide. We report two genotypes that have not been reported from Vietnam, in previous studies.

## Supporting Information

**Table S1.** RSV A and B reference sequences used in this study. **Table S2.** Identical sequences among nRSV and cRSV versus GenBank sequences. **Table S3.** The molecular characteristics of Vietnamese RSV A. **Table S4.** The molecular characteristics of the Vietnamese RSV B.
