# Supplementary material for: Characterization of hospital and community-acquired respiratory syncytial virus in children with severe lower respiratory tract infections in Ho Chi Minh City, Vietnam, 2010
Source: Influenza Other Respir Viruses. 2015 Apr 23;9(3):110–9. doi: 10.1111/irv.12307 (PMC4415695; doi:10.1111/irv.12307)
Supplement: Supplementary file 1 [file irv0009-0110-sd1.docx]

**Table S1: RSV A and B reference sequences used in this study**

| RSV A strains | | | RSV B strains | | |
| --- | --- | --- | --- | --- | --- |
| GenBank accession No. | Strain | Genotype | GenBank accession No. | Strain | Genotype |
| M11486 | A2 | Prototype | M17213 | CH18537 | Prototype |
| AF233914 | MO48 | GA1 | AF065250 | Ch10b | GB1 |
| AF233917 | NY108 | GA1 | M73542 | WV15291 | GB1 |
| AF065257 | CH34 | GA1 | AF233931 | NY01 | GB4 |
| AF233902 | AL19471-5 | GA1 | AF233924 | AL19734-4 | GB4 |
| AF233901 | AL19452-2 | GA6 | AF233928 | MO30 | GB4 |
| AF233918 | NY20 | GA6 | AF065251 | CH93/9b | GB2 |
| AB175815 | NG/009/02 | GA5 | AF348826 | SA98D1656 | SAB1 |
| AY472094 | Sal/173/99 | GA5 | AY524573 | Ken/109/02 | SAB1 |
| AF233903 | AL19556-3 | GA5 | AF348825 | SA0025 | SAB1 |
| AY146437 | Ab5076Pt01 | GA5 | AY751119 | BE/1252201 | BA2 |
| AY114150 | LLC24282 | GA5 | AY751123 | BE/1066/03 | BA2 |
| AF233906 | CN2708 | GA5 | DQ227377 | BA/1161/02 | BA2 |
| AF193310 | SE/10/92 | GA5 | DQ227391 | BA/4862/03 | BA2 |
| AF233919 | TX67591 | GA5 | AF309678 | Moz/205/99 | SAB2 |
| AF233909 | MO01 | GA5 | AF348821 | SA99V800 | SAB2 |
| AF348803 | SA97D1289 | GA5 | AF309684 | Moz/204/99 | SAB2 |
| AF348810 | SA98D707 | SAA1 | AY488800 | Mon/7/99 | SAB3 |
| AF348809 | SA97D606 | SAA1 | AY488795 | Mon/2/99 | SAB3 |
| AF348808 | SA99V1239 | SAA1 | GU550500 | Chongqing/B/09/23 | SAB4 |
| AF348807 | SA98V603 | SAA1 | GU550475 | Chongqing/B/06/02 | SAB4 |
| AF348804 | SA99V360 | GA7 | JN120007 | Cam2009-8166 | SAB4 |
| AF233904 | CN1973 | GA7 | JN119976 | Cam2009-0351 | SAB4 |
| AF233910 | MO02 | GA7 | AF233933 | TX69208 | GB3 |
| AF065254 | NY/CH09/93 | GA4 | AF233925 | AL19794-1 | GB3 |
| AF233920 | TX48481 | GA3 | AF233929 | MO35 | GB3 |
| AF233913 | MO16 | GA3 | DQ227368 | BA/166/02 | BA1 |
| AF233905 | CN2395 | GA3 | AY751131 | BE13417/99 | BA1 |
| AY472086 | Sal/87/99 | GA2 | AY333364 | BA4128/99B | BA1 |
| AF233915 | MO55 | GA2 | DQ227397 | BA/1607/04 | BA3 |
| AB470479 | NG-082-05 | NA2 | DQ227381 | BA/1441/02 | BA3 |
| FJ391425 | GER/0897/06-07 | NA1 | DQ227401 | BA/4825/03 | BA3 |
| DQ289605 | Beijing/A/04/07 | NA1 | AB175820 | NG/006/03 | BA5 |
| GU550471 | Chongqing/A/08/03 | NA1 | AB175819 | NG/004/03 | BA5 |
| FJ210838 | JU(IAL)1724/2007 | NA1 | AY751111 | BE/210/03 | BA6 |
| AB470478 | NG-016-04 | NA1 | AY751117 | BE/13159/02 | BA6 |
| JX079948 | 268-HCM | GA2? | DQ227401 | BA/4825/03 | BA6 |
| JX079949 | 378-HCM | GA2? | AY751094 | BE/12445/99 | BA4 |
| JX079959 | 688-HCM | GA2? | DQ227396 | BA/524/04 | BA4 |
|  |  |  | DQ227408 | BA/1526/04 | BA4 |
|  |  |  | AY751086 | BE/12670/01 | BA4 |
|  |  |  | HM459875 | NG-120-07 | BA8 |
|  |  |  | HM459873 | NG-013-07 | BA8 |
|  |  |  | AB603479 | NG-166-06 | BA8 |
|  |  |  | AB470482 | NG-064-06 | BA8 |
|  |  |  | AB603478 | NG-046-06 | BA8 |
|  |  |  | AB470481 | NG-013-05 | BA7 |
|  |  |  | HM459864 | NG-068-05 | BA7 |
|  |  |  | DQ227406 | BA/354/04 | BA7 |
|  |  |  | HM459890 | NG-017-07 | BA10 |
|  |  |  | HM459886 | NG-077-07 | BA10 |
|  |  |  | HM459887 | NG-047-07 | BA10 |
|  |  |  | HM459884 | NG-042-07 | BA10 |
|  |  |  | HM459883 | NG-084-07 | BA10 |
|  |  |  | AB603467 | NG-102-06 | BA9 |
|  |  |  | HM459876 | NG-022-06 | BA9 |
|  |  |  | DQ227395 | BA/100/04 | BA9 |
|  |  |  | HM459881 | NG-004-09 | BA9 |

**Table S2: Identical sequences among nRSV and cRSV versus GenBank sequences**

| Group | Identical sequences | | Identical sequences from GenBank (Strain and accession no.) |
| --- | --- | --- | --- |
|  | nRSV strains | cRSV strains |  |
| 1 | VN-202S2, VN-269S2, VN-432S1, VN-579S1, VN-5288S3, VN-5392S2 | 114 strains | Strain 797-HCM/11.10-A (JX079971)  Strain 894-HCM/10.10-A (JX079969)  Strain 846-HCM/10.10-A (JX079967)  Strain 753-HCM/09.10-A (JX079962)  Strain 657-HCM/08.10-A(JX079958)  Strain 597-HCM/07.10-A(JX079956)  Strain 512-HCM/06.10-A(JX079953)  Strain 415-HCM/05.10-A (JX079950)  Strain 378-HCM/05.10-A (JX079949) |
| 2 | VN-166S2 | VN-192 | Not found |
| 3 | VN-325S1 | VN-453, VN-457, VN-482, VN-517, VN-618, VN-5493, VN-5576, VN-5649 | Not found |
| 4 | VN-639S2 | VN-200, VN-297, VN-357, VN-370, VN-534, VN-606, VN-5217, VN-5361, VN-5446, VN-5496, VN-5534, VN-5544, VN-5550, VN-5561, VN-5646 | Strain 08-046972 (JX015498)  Strain BE/4998/08 (JX645856)  Strain 929-HCM/11.10-A (JX079970)  Strain 788-HCM/09.10-A (JX079964) |
| 5 | VN-5477S2 | VN-313, VN-334, VN-584, VN-698, VN-5379, VN-5409, VN-5469, VN-5475, VN-5484 | Identical to multiple sequences |
| 6 | VN-5503S3 | VN-223, VN-380, VN-385, VN-386, VN-5279, VN-5370, VN-5425, VN-5512 | Not found |
| 7 | VN-5517S1 | 0 | Not found |
| 8 | VN-5581S1 | 0 | Not found |

**Table S3: The molecular characteristics of Vietnamese RSV A**

| Genotype/Mutation | NA1 | | GA5 | Role | Reference |
| --- | --- | --- | --- | --- | --- |
|  | cRSV (N=262) | nRSV (N=13) | cRSV (N=5) |  |  |
| TTKP motif; 227-230 (%) | 96.2 | 92.3 | 100 | O-glycosylation site | ([5](#_ENREF_5), [17](#_ENREF_17), [19](#_ENREF_19), [20](#_ENREF_20)) |
| KPT motif; 233-235 (%) | 99.2 | 100 | 100 | O-glycosylation site |  |
| Asp237 (%) | 94.5 | 100 | 0 | NA1 specific  Loss of N-glycosylation site |  |
| Lys237(%) | 0.4 | 0 | 0 | Loss of N-glycosylation site |  |
| Ile238 (%) | 0 | 0 | 0 | GA5 specific |  |
| Pro241 | 0 | 0 | 100 | GA5 specific |  |
| TTKT motif; 238-241 (%) | 100 | 92.3 | 0 | O-glycosylation site |  |
| Asn250 | 0 | 0 | 100 | GA5 specific  Addition of N-glycosylation site |  |
| Ser251 | 0 | 0 | 100 | GA5 specific  Loss of N-glycosylation site |  |
| Asn251 | 88.2 | 76.9 | 0 | N-glycosylation site |  |
| Leu256 | 0 | 0 | 100 | GA5 specific |  |
| Thr269 | 100 | 100 | 0 | GA2 specific but also found in NA1 |  |
| Leu274 | 95.0 | 92.3 | 0 | NA1 specific |  |
| Thr274 | 0 | 0 | 100 | GA5 specific |  |
| Ile279 | 0 | 0 | 100 | GA5 specific |  |
| Ser289 | 100 | 100 | 0 | GA2 specific but also found in NA1 |  |
| Ser292 | 100 | 100 | 0 | NA1 specific |  |
| Tyr294 | 0 | 0 | 0 | Loss of N-glycosylation site |  |
| Ile295 | 0 | 0 | 100 | GA5 specific |  |
| Ile296 | 80.9 | 76.9 | 0 | Loss of N-glycosylation site at position 294 |  |
| Asp297 | 0 | 0 | 100 | GA5 specific |  |
| 298-STOP | 99.6 | 100 | 0 | NA1 specific |  |
| 299-STOP | 100 | 100 | 100 |  |  |

**Table S4: The molecular characteristics of the Vietnamese RSV B**

| Group/Genotype | | BA3 (N=3) | BA9 (N=26) | | BA10 (N=7) | Note | Reference |
| --- | --- | --- | --- | --- | --- | --- | --- |
|  |  |  | cRSV (N=25) | nRSV (N=1) |  |  |  |
| Predicted length (n) | 281 | 0 | 0 | 0 | 1 |  | ([5](#_ENREF_5)) |
|  | 312 | 1 | 25 | 1 | 3 |  |  |
|  | 315 | 2 | 0 | 0 | 0 |  |  |
|  | 319 | 0 | 0 | 0 | 3 |  |  |
| 20 aa duplication (n) | | 3 | 25 | 1 | 7 |  |  |
| Pro222 (n) | | 3 | 0 | 0 | 0 | Potential BA3 specific |  |
| NST; 230-232 (n) | | 0 | 0 | 0 | 2 | N-glycosylation site |  |
| Pro231 (n) | | 0 | 0 | 0 | 2 | GA10 specific  (230-232) |  |
| KPT; 234-236 (n) | | 3 | 25 | 1 | 7 | O -glycosylation site |  |
| Ile239 (n) | | 3 | 0 | 0 | 0 | Potential GA3 specific |  |
| Arg282 (n) | | 3 | 0 | 0 | 0 | Potential GA3 specific |  |
| Gly292 (n) | | 0 | 0 | 0 | 7 | GA10 specific |  |
| NST; 296-298 (n) | | 3 | 25 | 1 | 7 | N-glycosylation site |  |
| NST; 310-312 (n) | | 2 | 25 | 1 | 4 | N-glycosylation site |  |
